# Reducing Contrast Agent Dose in Cardiovascular MR Angiography with Deep Learning

**DOI:** 10.1002/jmri.27573

**Published:** 2021-02-22

**Authors:** Javier Montalt‐Tordera, Michael Quail, Jennifer A Steeden, Vivek Muthurangu

**Affiliations:** ^1^ Centre for Cardiovascular Imaging, UCL Institute of Cardiovascular Science University College London London WC1N 1EH UK; ^2^ Great Ormond Street Hospital London WC1N 3JH UK

**Keywords:** MR angiography, deep learning, contrast agent, low‐dose MRA

## Abstract

**Background:**

Contrast‐enhanced magnetic resonance angiography (MRA) is used to assess various cardiovascular conditions. However, gadolinium‐based contrast agents (GBCAs) carry a risk of dose‐related adverse effects.

**Purpose:**

To develop a deep learning method to reduce GBCA dose by 80%.

**Study Type:**

Retrospective and prospective.

**Population:**

A total of 1157 retrospective and 40 prospective congenital heart disease patients for training/validation and testing, respectively.

**Field Strength/Sequence:**

A 1.5 T, T1‐weighted three‐dimensional (3D) gradient echo.

**Assessment:**

A neural network was trained to enhance low‐dose (LD) 3D MRA using retrospective synthetic data and tested with prospective LD data. Image quality for LD (LD‐MRA), enhanced LD (ELD‐MRA), and high‐dose (HD‐MRA) was assessed in terms of signal‐to‐noise ratio (SNR), contrast‐to‐noise ratio (CNR), and a quantitative measure of edge sharpness and scored for perceptual sharpness and contrast on a 1–5 scale. Diagnostic confidence was assessed on a 1–3 scale. LD‐ and ELD‐MRA were assessed against HD‐MRA for sensitivity/specificity and agreement of vessel diameter measurements (aorta and pulmonary arteries).

**Statistical Tests:**

SNR, CNR, edge sharpness, and vessel diameters were compared between LD‐, ELD‐, and HD‐MRA using one‐way repeated measures analysis of variance with *post‐hoc t*‐tests. Perceptual quality and diagnostic confidence were compared using Friedman's test with *post‐hoc* Wilcoxon signed‐rank tests. Sensitivity/specificity was compared using McNemar's test. Agreement of vessel diameters was assessed using Bland–Altman analysis.

**Results:**

SNR, CNR, edge sharpness, perceptual sharpness, and perceptual contrast were lower (*P* < 0.05) for LD‐MRA compared to ELD‐MRA and HD‐MRA. SNR, CNR, edge sharpness, and perceptual contrast were comparable between ELD and HD‐MRA, but perceptual sharpness was significantly lower. Sensitivity/specificity was 0.824/0.921 for LD‐MRA and 0.882/0.960 for ELD‐MRA. Diagnostic confidence was 2.72, 2.85, and 2.92 for LD, ELD, and HD‐MRA, respectively (*P*_LD‐ELD_, *P*_LD‐HD_ < 0.05). Vessel diameter measurements were comparable, with biases of 0.238 (LD‐MRA) and 0.278 mm (ELD‐MRA).

**Data Conclusion:**

Deep learning can improve contrast in LD cardiovascular MRA.

**Level of Evidence Level:**

2

**Technical Efficacy:**

Stage 2

## Introduction

Assessment of vascular anatomy is one of the main indications for magnetic resonance (MR) in patients with congenital heart disease (CHD). Contrast‐enhanced MR angiography (CE‐MRA) is often used for imaging of the aortic and pulmonary vasculature and has a proven ability to detect vascular stenoses, dilation, and other abnormalities.^1^, ^2^, ^3^ The most commonly used contrast agents contain gadolinium chelates that reduce T1 and provide high contrast in T1‐weighted images. However, gadolinium‐based contrast agents (GBCA) have some potential adverse effects. Firstly, GBCAs can cause nephrogenic systemic fibrosis (NSF) in patients with renal disease,^4^, ^5^ which results in a poor prognosis. Secondly, the use of GBCAs results in organ deposition of gadolinium, particularly in the brain.^6^, ^7^, ^8^, ^9^ The clinical sequelae of organ deposition are poorly understood but are of increasing concern. Importantly, both problems are dose‐dependent, and there is great interest in lowering GBCA dose for routine CE‐MRA.^10^ An additional benefit from lowering the dose is cost reduction through less use of GBCA.

Unfortunately, low‐dose (LD) CE‐MRA results in poorer image quality,^10^ which may affect diagnostic utility. It has been shown that a deep learning postprocessing step can recover high‐dose (HD) CE‐MRA characteristics from LD brain MRA images.^11^ This study aims to investigate whether a similar approach can be used to regain HD features from LD‐MRA of the great vessels in CHD.

## Materials and Methods

This study was approved by the local research ethics committee (Ref. 06/Q0508/124), and written consent was obtained from all subjects or guardians in prospective and retrospective cohorts.

### Prospective Cohort and Imaging

Forty children and adults with CHD were recruited for this study. The inclusion criterion was a clinical referral for cardiovascular magnetic resonance imaging (MRI) including CE‐MRA. The only exclusion criterion was the inability to breath‐hold (two patients excluded). MRA images were acquired with a 1.5 T scanner (Avanto, Siemens Healthineers AG, Erlangen, Germany) using a Cartesian three‐dimensional (3D) gradient echo (GRE) sequence with the following parameters: matrix size = 256 × (128–172) × (96–160), voxel size = 1.6 × 1.6 × 1.6 mm^3^, TE/TR = 1.0/3.0 msec, and sagittal orientation. Imaging was timed with respect to injection of the GBCA bolus to obtain either aortic angiograms or pulmonary angiograms (20 subjects each). Average acquisition time per MRA image was 14.2 ± 2.03 seconds.

**Figure 1:**
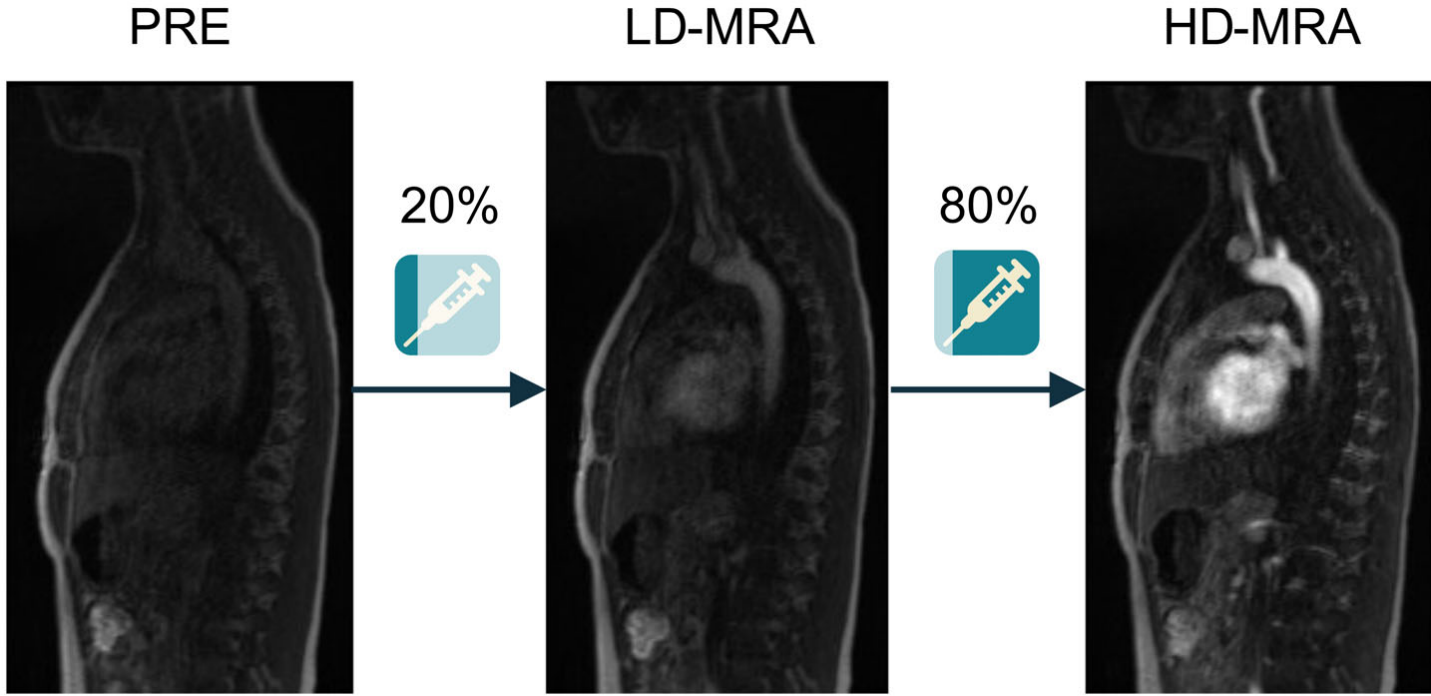
Acquisition protocol for a prospective clinical study. Three images were acquired for each subject: pre‐contrast (PRE), low‐dose (LD‐MRA) after injection of 20% of dose, and high‐dose (HD‐MRA) after injection of the remaining dose.

Each subject was administered a total of 0.2 mmol/kg bodyweight of gadoteric acid (Dotarem, Guerbet, Villepinte, France) up to 10 mmol, which is the normal GBCA dose at our institution. Two CE‐MRA images were collected for each patient by splitting the dose in two (Fig. 1). 20% of the dose (0.04 mmol/kg, up to 2 mmol) was injected first to obtain a LD angiogram (LD‐MRA), followed by the remaining 80% (0.16 mmol/kg, up to 8 mmol) to obtain a HD angiogram (HD‐MRA). The HD‐MRA was performed immediately after the LD‐MRA so that the low dose contrast was still in circulation, resulting in an angiogram that approximated to 100% dose.

**Figure 2:**
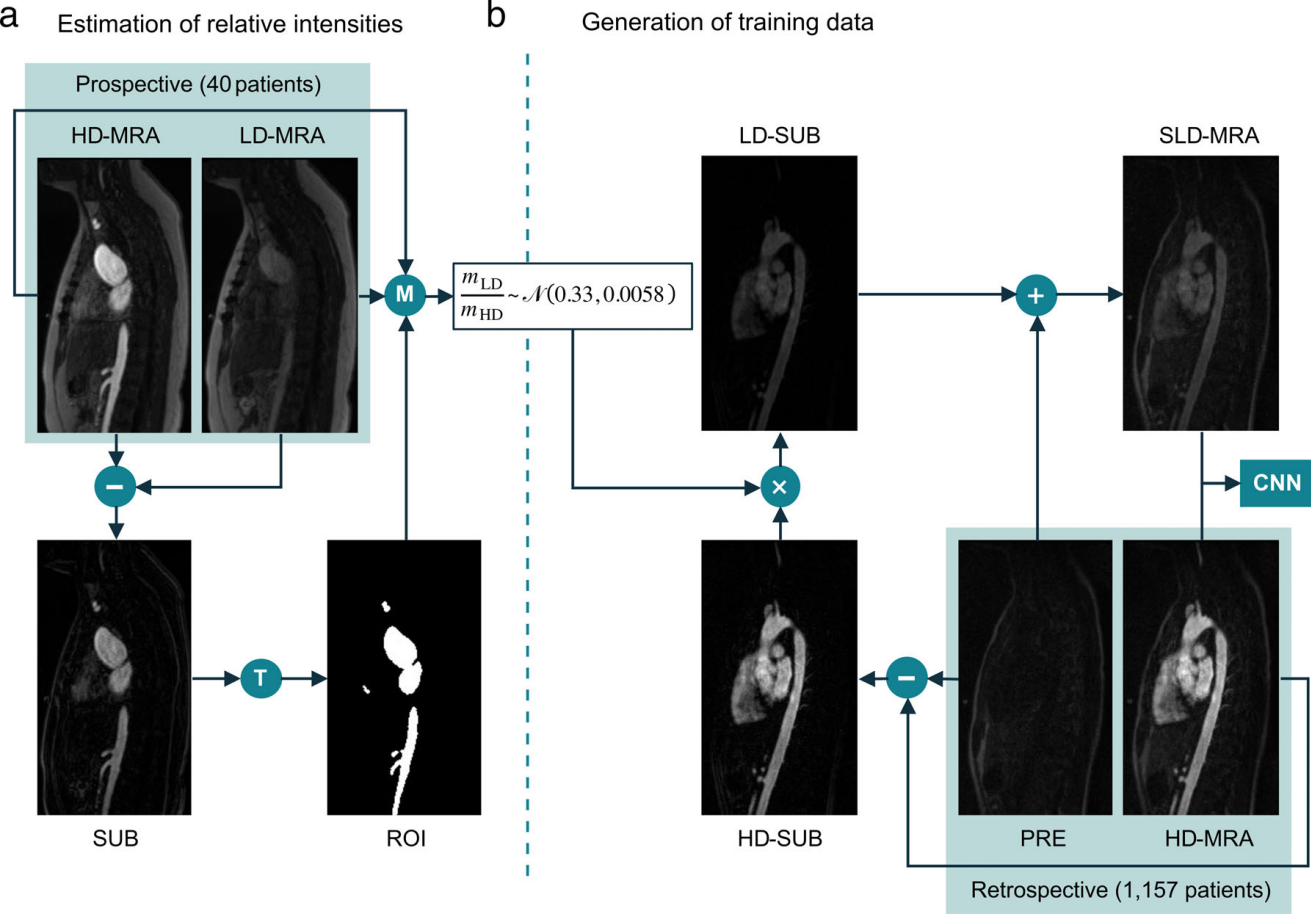
Preparation of synthetic training data. (A) Estimation of the intensity ratio, *R*, between prospective low‐dose (LD‐MRA) and high‐dose (HD‐MRA) images. T: thresholding followed by morphological opening. M: compute mean over ROI for both images. (B) Generation of synthetic low‐dose (SLD‐MRA) images, using *R*, to be paired with the corresponding high‐dose (HD‐MRA) images to train the convolutional neural network (CNN).

### Retrospective Cohort and Imaging

Conventional CE‐MRA images were retrieved from all patients who had undergone clinical aortic or pulmonary CE‐MRA examinations at our institution between 2017 and 2018. Images with poor quality due to respiratory motion, patient motion, or poor contrast timing were excluded (50 patients). A total of 1173 CE‐MRA examinations were obtained from CHD patients aged 25 ± 16 years (range: 0–76). Of these, 663 were aortic angiograms and 510 were pulmonary angiograms. Each CE‐MRA examination consisted of a pre‐contrast image, acquired before any GBCA injection, and a post‐contrast image, acquired after injection of contrast bolus with a high dose (HD‐MRA). The sequence type and imaging parameters were the same as described for the prospective cohort.

### Preparation of Synthetic Training Data

The retrospective HD‐MRA data were used to simulate a corresponding synthetic LD‐MRA (SLD‐MRA) dataset (Figure 2B) to train the neural network. First, a difference image was calculated by subtracting the pre‐contrast image from the post‐contrast image. The resulting image, which reflects primarily the GBCA signal, was multiplied by a factor *R*, which was computed from the prospective data. Specifically, *R* was calculated as the ratio of average pixel intensity in contrast‐enhancing tissue from LD‐MRA (*m*_LD_) to HD‐MRA (*m*_HD_) in regions of interest (ROIs) computed automatically by thresholding the difference between both images, using Otsu's method^12^ (Figure 2A). The scaled image was added back to the pre‐contrast image to generate the post‐contrast SLD‐MRA image. Finally, pixel intensities were rescaled to the range [0, 1] using min–max normalization. This resulted in 1173 pairs of SLD‐MRA and HD‐MRA images.

### Network Architecture

A variant of the U‐Net^13^ architecture was used in this study. Following the principle of residual learning,^14^, ^15^ a global residual skip connection was added to learn the residual with respect to the input image rather than the output image itself.

The residual U‐Net was implemented with four encoding and four decoding steps. Each encoding/decoding step contained two 3D convolutional layers with 3 × 3 × 3 kernels, each followed by rectified linear unit (ReLU) activations. Encoding steps additionally included a max‐pooling layer to perform 2 × 2 × 2 downsampling, while decoding steps included a transpose convolution with stride 2 × 2 × 2 to perform upsampling. The first convolutional layer had 32 filters, and this number doubled with each subsequent encoding step up to a maximum of 512 channels. Each decoding step then halved the number of filters back to 32. A final bottleneck layer (1 × 1 × 1 convolution with one filter) was added to combine all features into a single channel, followed by the residual addition and a ReLU activation.

### Network Training and Validation

To prepare for training, the synthetic dataset was randomly split into a training set (90%, 1056 HD‐MRA – SLD‐MRA pairs) and a validation set (10%, 117 pairs). These were padded/cropped to matrix size 192 × 128 × 112 (superior–inferior, anterior–posterior, left–right). These dimensions were deemed sufficient to cover the anatomy of interest in the majority of patients. While the model is agnostic to image size, training was observed to be significantly faster when using a fixed input size.

The neural network was implemented and trained in TensorFlow (Google LLC, Mountain View, CA, USA). The network weights were initialized using He′s method^16^ and trained by minimizing the mean squared error (or *ℓ*_2_ loss) between network outputs and ground truth images. The Adam algorithm,^17^ with an initial learning rate of 10^−3^, was used for the optimization. Training continued for 60 epochs, in batches of two volumes. Validation loss in our model was seen to stabilize within 60 epochs. Training took 36 hours on an Nvidia Titan RTX GPU with 24GB of onboard RAM (Nvidia Corporation, Santa Clara, CA, USA). Performance was evaluated on the validation set, unseen by the network, using structural similarity index (SSIM) and peak signal‐to‐noise ratio (PSNR) metrics.

### Network Testing

The trained neural network was used to enhance contrast in LD‐MRA images acquired in the prospective patient cohort. We refer to the network output as ELD‐MRA (enhanced LD‐MRA). Processing time per volume on the GPU (Titan RTX 24 GB) was recorded.

All image analysis was performed using the open‐source software Horos DICOM Medical Image Viewer (Horos v4.0, Horos Project) with in‐house plugins. In all analyses, observers were blinded to image type and were presented with anonymized data in random order.

#### VESSEL SEGMENTATION AND DIAMETER MEASUREMENTS

Cross‐sectional images of the great vessels were obtained using multiplanar reformation (MPR) from LD, ELD, and HD‐MRA volumes. The specific vessels were the ascending aorta (AAO) and descending aorta (DAO) for aortic angiograms; and main pulmonary artery (MPA), left pulmonary artery (LPA), and right pulmonary artery (RPA) for pulmonary angiograms.

Using the MPR images, an imaging cardiologist (V.M., 17 years of experience, primary observer) manually measured the diameter of each vessel for each patient and image type (LD, ELD, and HD‐MRA). For each vessel, two measurements were taken in perpendicular directions, and the average was used for further analysis. A subset of 10 randomly selected patients (five aortic angiograms and five pulmonary angiograms) were re‐evaluated by the primary observer to assess intra‐observer variability. This subset was also evaluated by a second imaging cardiologist (M.Q., 12 years of experience, secondary observer) to assess inter‐observer variability.

#### EVALUATION OF IMAGE QUALITY

Image quality of LD, ELD, and HD‐MRA images was assessed according to three objective metrics, namely estimated signal‐to‐noise ratio (SNR), estimated contrast‐to‐noise ratio (CNR), and vessel edge sharpness; and two subjective scores: perceptual sharpness and perceptual contrast.

Quantitative SNR and CNR were assessed in a mid‐thoracic axial slice cutting across the vessel of interest (aorta or pulmonary artery). Two elliptical regions of interest were manually delineated (J.M.T., 3 years of experience): one in the vessel and one in the spinal canal, a tissue that has a low signal and is non‐contrast enhancing. The regions were drawn to cover the largest possible area while remaining within the edges of the relevant structure. Signal was estimated as the average intensity in the vessel (*m*_vessel_), contrast was estimated as the difference between the average intensity in the vessel and the average intensity in the spinal canal, and noise was estimated as the standard deviation in the spinal canal. Therefore:

$$\mathrm{SNR}=\frac{m_{\text{vessel}}\;}{s_{\text{tissue}}}$$

$$\mathrm{CNR}=\frac{m_{\text{vessel}}-m_{\text{tissue}}}{s_{\text{tissue}}}$$

Quantitative edge sharpness was estimated on the MPR data (all vessels) by measuring the maximum gradient of the normalized pixel intensities across the edge of the vessel of interest, as has been described previously.^18^ The measurement was performed in an automated fashion in 60 uniformly spaced positions around each vessel. Outliers, defined as those values beyond three scaled mean absolute deviations from the median, were removed to improve robustness. The remaining values were averaged for comparison.

Perceptual quality scores were assessed by three observers (V.M., 17 years of experience; M.Q., 12 years of experience; J.A.S., 13 years of experience) on all MPR images (LD, ELD, and HD‐MRA) using a 5‐point Likert scale (1 = nondiagnostic, 2 = poor, 3 = adequate, 4 = good, 5 = excellent) in two categories: sharpness of vessel borders and vessel contrast. The observer was blinded to image type and was presented with the images in random order.

#### DIAGNOSTIC ACCURACY AND CONFIDENCE

Identification of abnormal anatomy was performed on LD, ELD, and HD‐MRA images by consensus of two imaging cardiologists (V.M., 17 years of experience; and M.Q., 12 years of experience). Observers were presented with the full 3D data. For each volume, the observers were asked to identify the presence of the following conditions, depending on angiogram type. For aortic angiograms, possible conditions were: 1) coarctation of the aorta, 2) aortic dilatation, and 3) abnormal arch anatomy. For pulmonary angiograms, they were: 1) MPA stenosis, 2) LPA stenosis, and 3) RPA stenosis. In each case, the observer rated the likelihood that the abnormality is present on a 5‐point Likert scale (1 = definitely not present, 2 = probably not present, 3 = unclear, 4 = probably present, and 5 = definitely present). For the purposes of diagnostic accuracy evaluation, the diagnosis was considered negative (condition absent) if the score was 1 or 2 and positive (condition present) if the score was 4 or 5. A score of 3 was considered a misdiagnosis. Sensitivity and specificity were computed for LD and ELD‐MRA diagnoses, using HD‐MRA images as the reference standard. For the purposes of diagnostic confidence evaluation, observer responses were classified into three confidence levels: low or “unclear if condition present” (score of 3), intermediate or “condition probably (not) present” (score of 2 or 4), and high or “condition definitely (not) present” (score of 1 or 5). In turn, these confidence levels were coded as 1, 2, and 3, respectively, for quantification and comparison.

### Statistical Analysis

Validation metrics SSIM and PSNR for synthetic LD images and their enhanced counterparts were compared using a paired *t*‐test. For the prospective cohort, SNR, CNR, edge sharpness, and vessel diameters (continuous and normally distributed) were compared across all three image types using one‐way repeated measures analysis of variance (ANOVA). Significant results were followed up by *post hoc* pairwise paired *t*‐tests with Holm correction to determine pairwise significant differences. Perceptual quality scores and diagnostic confidence (Likert scale, discrete, and ordinal) were compared across image types using Friedman's test, followed by *post hoc* Wilcoxon signed‐rank tests with Holm correction. Vessel diameters were grouped by vessel and compared using one‐way repeated measures ANOVA. In addition, agreement between LD‐MRA versus HD‐MRA measurements and ELD‐MRA versus HD‐MRA measurements was assessed using Bland–Altman analysis. The diagnostic accuracy (sensitivity and specificity) between LD‐MRA and ELD‐MRA images was compared using McNemar's test. In addition to the overall tests, SNR, CNR, edge sharpness, perceptual quality scores, diagnostic accuracy, and diagnostic confidence were also assessed separately for aortic and pulmonary angiograms. Intra‐ and inter‐observer agreement in diameter measurements were assessed using the intraclass correlation coefficient (ICC) for a two‐way random‐effects model. The agreement was considered poor for ICC < 0.5, moderate for 0.5 < ICC < 0.75, good for ICC 0.75 < ICC < 0.9, and excellent for ICC > 0.9. All statistical analysis was performed using *R*.^19^ Test results were considered statistically significant if the *P*‐value was smaller than 0.05.

## Results

### Network Validation

The intensity ratio *R* in the prospective cohort was found to be approximately normally distributed with mean 0.331 and variance 5.84 × 10^−3^.

**Figure 3:**
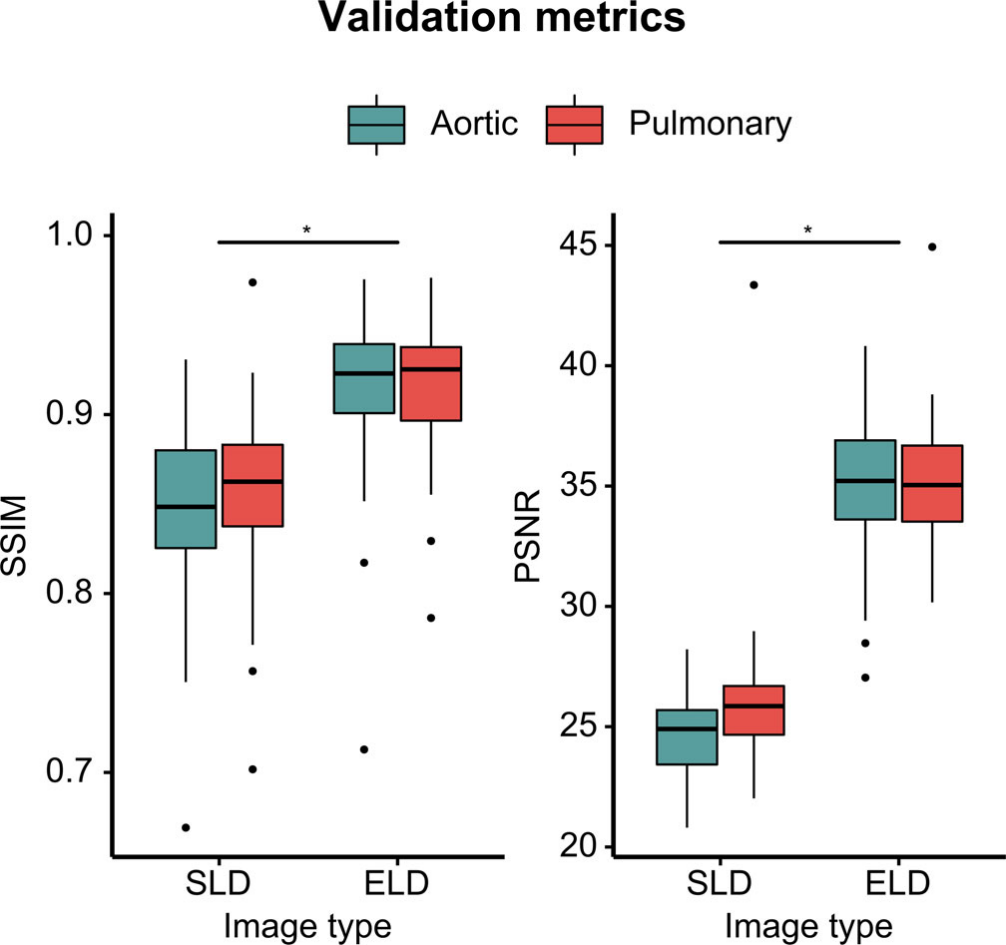
Validation metrics in synthetic dataset. SSIM: structural similarity index; PSNR: peak signal‐to‐noise ratio; SLD: synthetic low‐dose; ELD: enhanced low‐dose. *P < 0.05.

**Figure 4:**
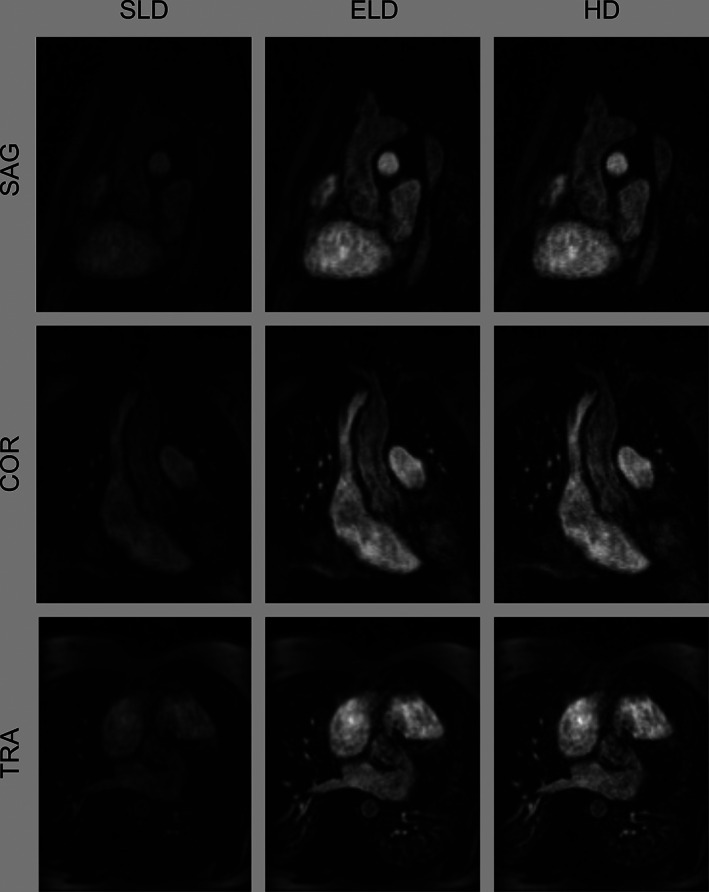
Representative images from a subject in the validation set. Sagittal and coronal views are cropped to the anatomy of interest. SLD: synthetic low‐dose; ELD: enhanced low‐dose; HD: high‐dose; SAG: sagittal; COR: coronal; TRA: transverse.

The SLD‐MRA in the validation set had an SSIM of 0.852 ± 0.046 and a PSNR of 25.3 ± 2.35 with respect to the original HD‐MRA data. After enhancement by the neural network (ELD‐MRA), SSIM and PSNR significantly increased (*P* < 0.05 for both) to 0.914 ± 0.037 and 35.2 ± 2.54, respectively (Figure 3). Representative examples in different anatomical orientations are shown in Figure 4.

### Network Testing

The prospective cohort had the following demographics: age 26.7 ± 13 years (range 13–62), 16 female, 24 male. The clinical diagnoses were: repaired tetralogy of Fallot/pulmonary atresia ventricular septal defect (VSD) (*n* = 8), transposition of the great arteries post ASO (*n* = 5), Ebstein's anomaly (*n* = 3), VSD/atrioventricular septal defect (*n* = 4), pulmonary hypertension (*n* = 1), Marfan syndrome/aortopathy (*n* = 7), bicuspid aortic valve (*n* = 7), cardiomyopathy/myocarditis (*n* = 3), and repaired coarctation of the aorta (*n* = 3).

**Figure 5:**
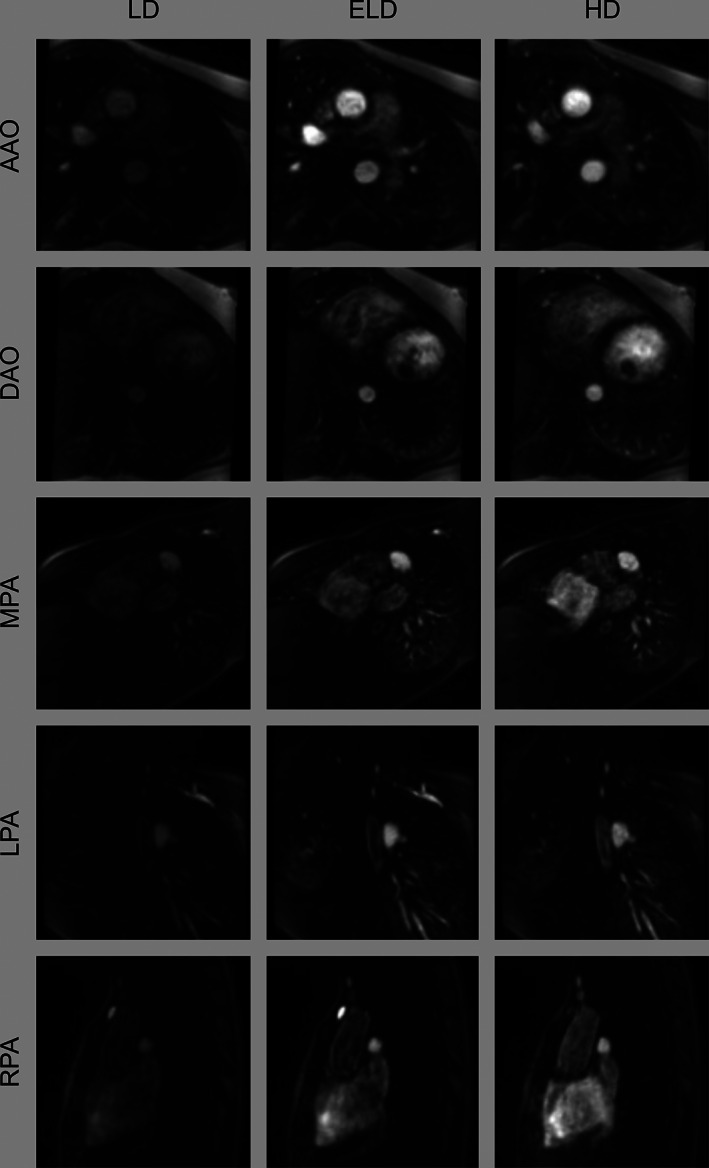
Representative images of vessels from the prospective study. Multiplanar reformats of the ascending aorta (AAO), descending aorta (DAO), main pulmonary artery (MPA), left pulmonary artery (LPA), and right pulmonary artery (RPA). LD: low‐dose; ELD: enhanced low‐dose; HD: high‐dose.

Processing time to enhance the LD images using the trained neural network was approximately 1.1 s per volume. Representative examples of LD, ELD, and HD images depicting the various vessels of interest are shown in Figure 5.

#### EVALUATION OF IMAGE QUALITY

**Figure 6:**
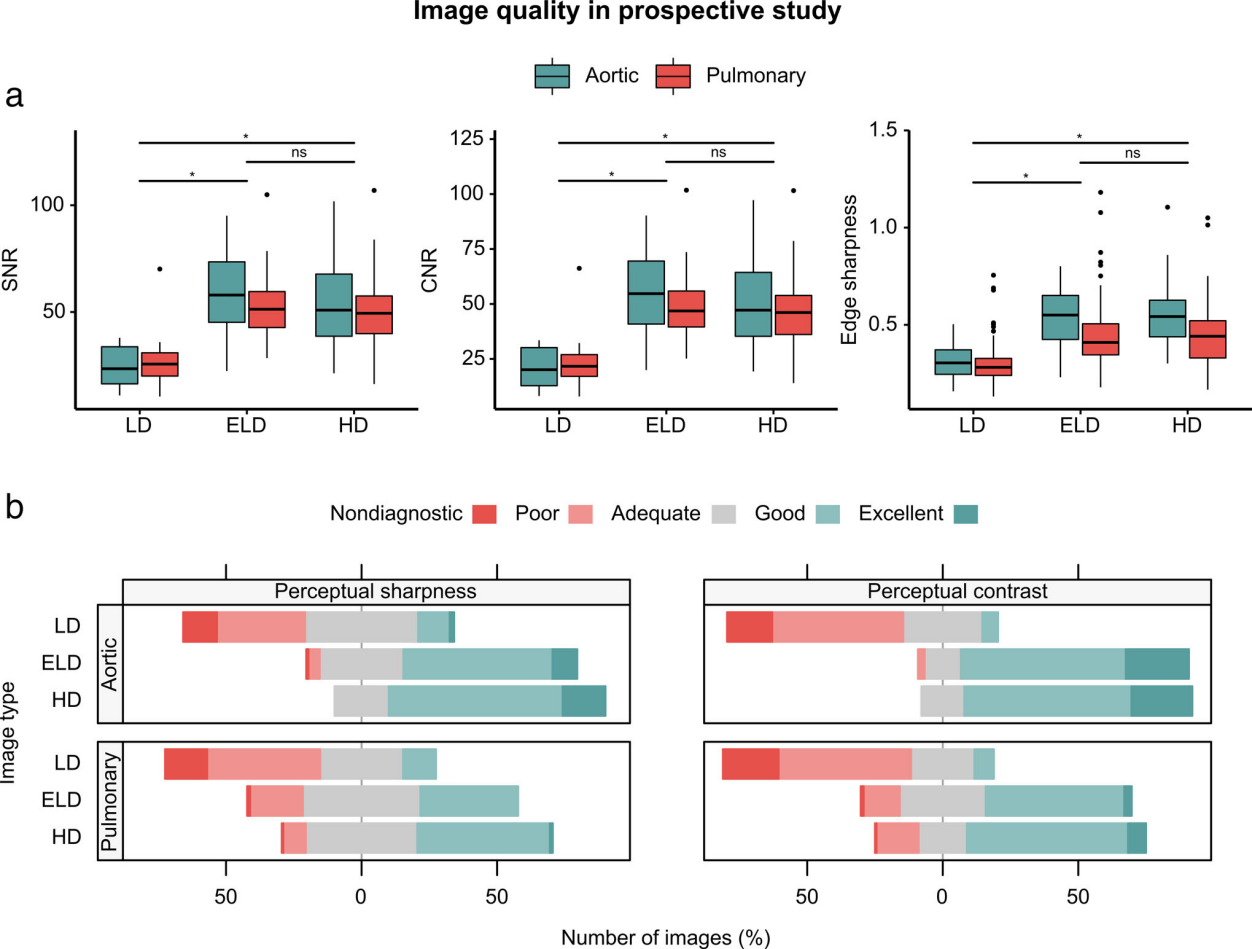
Image quality in a prospective study. (A) Objective image quality metrics: signal‐to‐noise ratio (SNR), contrast‐to‐noise ratio (CNR), and edge sharpness. (B) Perceptual image quality in terms of sharpness and contrast, as rated by three observers. LD: low‐dose; ELD: enhanced low‐dose; HD: high‐dose; ns: nonsignificant; *: *P* < 0.05.

Objective and perceptual image quality metrics are summarized in Figure 6. SNR, CNR, edge sharpness, perceptual sharpness, and perceptual contrast were found to be significantly lower (*P* < 0.05) for LD‐MRA images (25.7 ± 10.9, 22.0 ± 10.6, 0.309 ± 0.108, 2.47 ± 0.91 and 2.20 ± 0.82, respectively) compared to ELD‐MRA images (56.5 ± 19.7, 52.4 ± 19.2, 0.492 ± 0.176, 3.36 ± 0.80 and 3.68 ± 0.80, respectively) and HD‐MRA images (53.6 ± 22.4, 50.0 ± 21.8, 0.493 ± 0.176, 3.63 ± 0.70 and 3.76 ± 0.81, respectively). No significant differences could be found between ELD‐MRA images and HD‐MRA images for SNR, CNR, edge sharpness, or perceptual contrast (*P* = 0.483, *P* = 0.533, *P* = 0.930, and *P* = 0.132, respectively). However, a statistically significant difference was found between ELD‐MRA and HD‐MRA for perceptual sharpness (*P* < 0.05). Metrics and *P*‐values for aortic and pulmonary angiograms are given in Supplementary Table S1. When aortic and pulmonary angiograms were considered separately, SNR, CNR, edge sharpness, perceptual sharpness, and perceptual contrast were also found to be significantly lower for LD‐MRA compared to ELD‐MRA and HD‐MRA. No statistically significant differences were found in SNR, CNR, edge sharpness, or perceptual contrast between ELD and HD‐MRA images for either aortic or pulmonary angiograms. A statistically significant difference was found in perceptual sharpness between ELD and HD‐MRA images in both aortic and pulmonary angiograms.

#### DIAGNOSTIC ACCURACY AND CONFIDENCE

**Figure 7:**
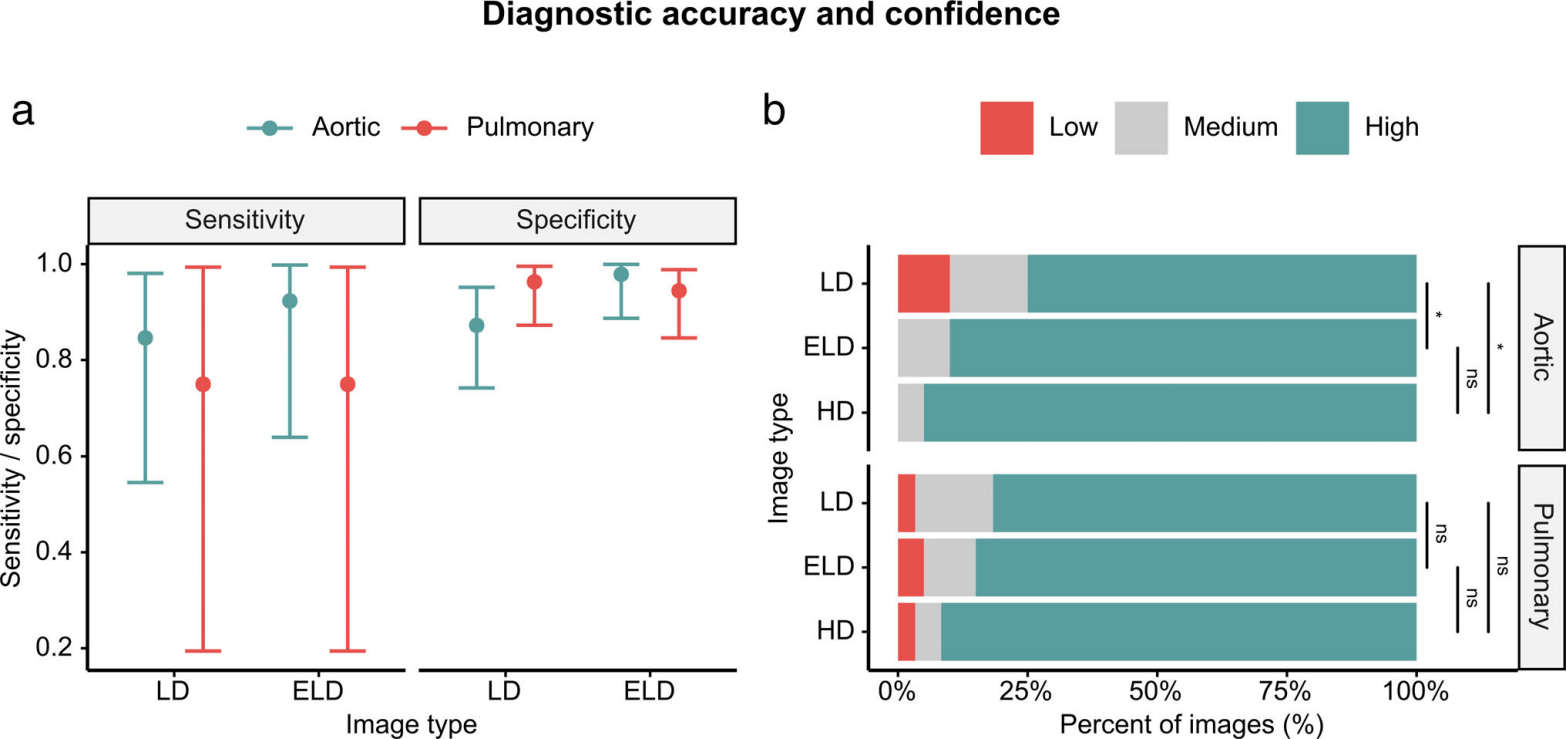
Diagnostic accuracy and confidence. (A) Sensitivity and specificity for the detection of lesions in LD and ELD images with 95% confidence intervals. (B) Confidence expressed in diagnosis based on LD, ELD, and HD images. Confidence levels: low, “unclear if condition present”; medium, “condition probably (not) present”; high, “condition definitely (not) present.” LD: low‐dose; ELD: enhanced low‐dose; HD: high‐dose; ns: nonsignificant; *: *P* < 0.05.

The sensitivities and specificities of LD and ELD‐MRA are summarized in Figure 7A. Overall sensitivity was 0.824 (95% confidence interval (CI): 0.566–0.962) for LD‐MRA and 0.882 (95% CI: 0.636–0.985) for ELD‐MRA. Overall specificity was 0.921 (95% CI: 0.850–0.965) for LD‐MRA and 0.960 (95% CI: 0.902–0.989) for ELD‐MRA. These differences were not statistically significant (*P* = 0.546). Sensitivity, specificity, and *P*‐values by angiogram and lesion type are reported in Supplementary Table S2. There were no statistically significant differences between LD and ELD‐MRA in either aortic angiograms or pulmonary angiograms.

Diagnostic confidence is summarized in Figure 7B. Confidence was 2.72 ± 0.582 for LD‐MRA, 2.85 ± 0.423 for ELD‐MRA, and 2.92 ± 0.333 for HD‐MRA. Statistically significant differences (*P* < 0.05) were found between LD and ELD‐MRA and between LD and HD‐MRA, but no statistically significant difference was found between ELD and HD‐MRA (*P* = 0.064). Diagnostic confidence values and *P*‐values by angiogram and lesion type are reported in Supplementary Table S3. In aortic angiograms, there were significant differences between LD and ELD‐MRA and between LD and HD‐MRA, but not between ELD and HD‐MRA. In pulmonary angiograms, there were no significant differences between any of the groups.

#### VESSEL DIAMETER MEASUREMENTS

There were no statistically significant differences in diameters measured from LD, ELD, and HD‐MRA images for any vessel (overall: *P* = 0.110, AAO: *P* = 0.388, DAO: *P* = 0.167, MPA: *P* = 0.079, LPA: *P* = 0.744, RPA: *P* = 0.171). Overall bias was 0.238 mm (limits of agreement: −2.93 – 3.41) for LD‐MRA and 0.278 mm (−2.71–3.26) for ELD‐MRA.

**Figure 8:**
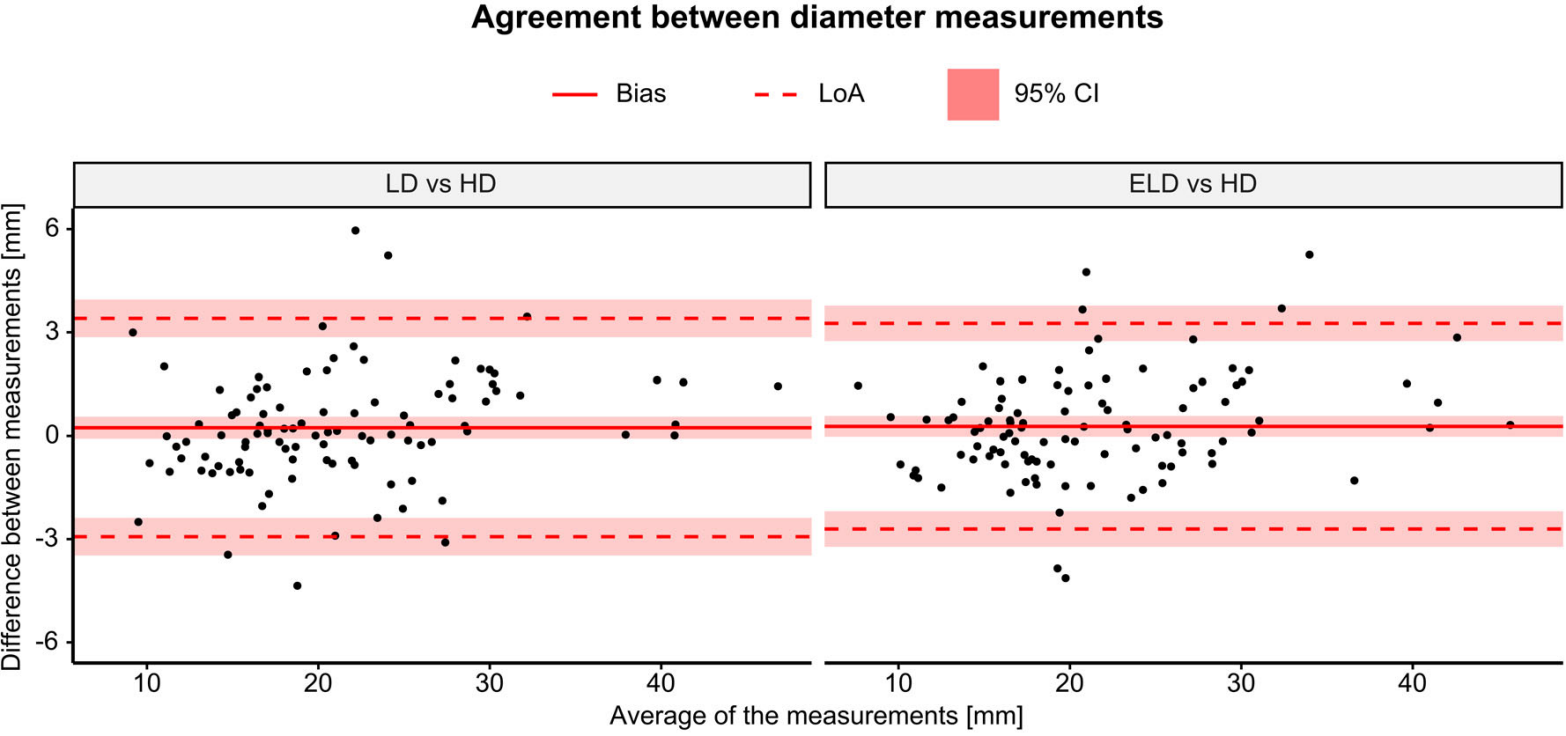
Bland–Altman plots of agreement between diameter measurements. LD: low‐dose; ELD: enhanced low‐dose; HD: high‐dose; LoA: limits of agreement; CI: confidence interval.

Table 1 summarizes the vessel diameters and the results of the Bland–Altman analysis for each individual vessel. Figure 8 shows Bland–Altman plots of agreement between LD and HD‐MRA and between ELD and HD‐MRA, for all vessels combined. Bland–Altman plots for individual vessels are shown in Figure S1.

**Table 1:** Vessel diameter measurements and Bland–Altman analysis

Vessel	*N*	Diameter (mm)			Bland–Altman (mm)		*P*‐Value
		LD	ELD	HD	LD	ELD	
AAO	20	29.7 ± 7.92	29.4 ± 7.68	29.3 ± 7.36	0.377 (−2.49–3.24)	0.137 (−2.40–2.67)	0.388
DAO	20	17.0 ± 2.23	17.2 ± 2.02	17.4 ± 2.00	−0.348 (−2.36–1.66)	−0.189 (−1.52–1.14)	0.167
MPA	20	25.8 ± 6.45	26.4 ± 7.02	25.5 ± 6.27	0.333 (−3.61–4.27)	0.928 (−2.86–4.72)	0.079
LPA	20	16.4 ± 4.54	16.2 ± 4.41	16.1 ± 4.76	0.263 (−3.15–3.67)	0.057 (−3.33–3.44)	0.744
RPA	20	17.9 ± 4.00	17.8 ± 3.69	17.3 ± 3.65	0.567 (−2.73–3.87)	0.455 (−2.60–3.51)	0.171
All	100	21.4 ± 7.56	21.4 ± 7.60	21.1 ± 7.33	0.238 (−2.93–3.41)	0.278 (−2.71–3.26)	0.110

*Note*: Diameters are reported in mm as mean ± SD and Bland–Altman results are reported as bias and limits of agreement.

AAO: ascending aorta; DAO: descending aorta; ELD: enhanced low dose; HD: high dose; LD: low dose; LPA: left pulmonary artery; MPA, main pulmonary artery; RPA: right pulmonary artery.

Inter‐observer agreement was excellent with ICCs of 0.987 (95% CI: 0.971–0.994) for LD‐MRA, 0.986 (95% CI: 0.957–0.994) for ELD‐MRA and 0.986 (95% CI: 0.957–0.994) for HD‐MRA. Intra‐observer agreement was also excellent with ICCs of 0.983 (95% CI: 0.961–0.993), 0.993 (95% CI: 0.983–0.997), and 0.993 (95% CI: 0.954–0.998), respectively.

## Discussion

The main findings of our study were 1) the trained CNN we used was able to recover image contrast in synthetic and prospectively acquired LD angiograms, 2) the image quality of the enhanced LD angiograms was superior to the original LD data, 3) diagnostic confidence for enhanced images was comparable to HD images and significantly better than in LD images, and 4) there were no differences in diagnostic accuracy or vessel measurements between the enhanced and original low dose angiograms.

LD‐MRA reduces risk from GBCAs,^10^ which is particularly pertinent in pediatric and CHD, due to the need for life‐time imaging follow‐up.^20^ LD angiography could also be useful in patients with renal disease, who are at risk of developing NSF with the administration of high doses of GBCA.^4^, ^5^ However, as we have shown, LD‐MRA suffers from poorer image quality.

We have shown that our method can improve image quality in LD‐MRA. Nevertheless, even though LD‐MRA had lower image quality, sensitivity and specificity were not significantly different from ELD‐MRA. This is in keeping with other studies that have indicated that LD‐MRA has similar diagnostic accuracy to HD‐MRA.^21^, ^22^ In our study, one possible reason for this finding is that experienced imaging specialists are able to identify abnormalities even when image quality is poor. However, this requires formal investigation by comparing diagnostic accuracy in observers with very different levels of experience. Nevertheless, diagnostic confidence did improve significantly after deep learning enhancement. This may be important in clinical practice, as higher confidence may reduce the need for further testing and lead to prompter responses.

Another important use of angiographic data is the measurement of vessel diameters. Manual vessel diameter measurement in HD‐MRA is the current clinical standard and we found that vessel diameter measurements taken from either LD or enhanced LD images agreed well with the reference HD measurements. This suggests that the proposed method is true to the underlying anatomy and that reliable measurements can be extracted from enhanced LD images.

In this study, we chose to use an architecture based on the U‐Net, which was originally introduced for semantic segmentation.^13^ U‐Net‐like architectures have become popular and have demonstrated good performance in a variety of problems including image reconstruction and processing.^23^, ^24^, ^25^ Their power seems to lie in the ability to integrate high‐level semantic information with spatial information.^26^ We believe that enhancing contrast also requires these characteristics and that U‐Nets are well suited to this problem.^13^ Indeed, a U‐Net has previously been used to enhance contrast in LD brain MRI.^11^ In addition, processing with a U‐Net is fast and can be easily incorporated into the clinical workflow as part of the reconstruction pipeline or as an extension to medical imaging visualization software.

In general, neural network performance is expected to improve with the amount of training data.^27^ Collecting a large prospective dataset for our study would be technically possible, but it would be a complex and costly endeavor. In addition, prospectively acquired image pairs would be subject to misregistration and differences in contrast timing that would impair mapping between the two. Instead, our approach used routinely acquired HD images and their pre‐contrast counterparts to simulate equivalent LD angiograms. This is in contrast with a previous study on deep learning‐enhanced LD brain MRA, which used a smaller prospectively acquired dataset for training.^11^

Recently, Ferumoxytol has found off‐label use as an MRI contrast agent and is a promising nonnephrotoxic alternative to GBCAs in CHD.^28^, ^29^ However, Ferumoxytol is not available for use as a contrast agent in many parts of the world (i.e., Europe). Thus, we believe that our work still has relevance from a global point of view. In addition, our approach may also be useful for Ferumoxytol angiography. Firstly, dose reduction may enable simpler administration without the need for long infusion times.^30^ Secondly, Ferumoxytol is more expensive than GBCAs (~$100/vial vs. $900/vial),^30^ so the relative cost reduction associated with Ferumoxytol would be greater than with GBCAs. Future studies could investigate the feasibility of using our network for Ferumoxytol MRA, perhaps optimized with a transfer learning step.

### Limitations

While we did not observe any inaccuracies in our test data, one concern is the risk of reduced accuracy on rarer congenital heart defects, which may be underrepresented in the training data. In addition, our population was limited to patients with CHD as this is the specialization of our MR service. However, our training dataset contained more than 1000 images with significant variability in anatomies and conditions, which should mitigate the risk of poor generalizability. Increasing the size of this dataset is possible and could further improve the robustness of the method. In addition, the network is residual and only generates sparse feature differences, reducing the potential for hallucination.^31^ Finally, we believe that the network is more likely to rely on general features than specific anatomies, as previously suggested in networks with related architectures.^23^ Nevertheless, future work is required to expand the diagnoses of the patient population and evaluate different vessels such as coronary and renal arteries, as well as patients with implanted devices. Another possible limitation is the experimental design of the prospective data acquisition, where two angiograms were acquired during the same examination. The second angiogram was acquired after injection of a bolus containing 80% of the dose, the remaining 20% having been given a few minutes earlier. This may result in slightly different contrast dynamics than after a single 100% bolus. However, the alternative experimental design imposes important costs. First, it would require the patient to come to the institution twice. With a half‐life of 1.5 hours in healthy subjects (longer in patients with renal impairment), the contrast agent is not cleared to negligible levels until at least the next day.^32^ Second, it would involve administering the patient a cumulative 120% dose, higher than otherwise required for their standard care. We believe that the additional accuracy does not justify these costs. Therefore, the current design, which has been similarly used before,^11^ was preferred.

Another issue was that there was a low prevalence of lesions in our prospective patient population. This limited our ability to infer differences in diagnostic accuracy (particularly sensitivity) between LD and enhanced angiograms. Thus, a larger study enriched with more true positives is needed to definitively assess diagnostic accuracy. Such a study could be further improved using data from multiple scanner types and by including less experienced observers.

Finally, throughout this study we used a fixed dose of GBCA, set to 20% of the normal dose. However, the enhancement method performed well and LD images often had better quality than expected. Therefore, we believe that even lower doses might be possible. Indeed, another study on deep learning enhancement for brain MRA used a 10% dose.^11^ Other non‐MRA studies have attempted to completely eliminate the use of a contrast agent,^33^, ^34^ though whether this would be possible in MRA is unclear. Nevertheless, an 80% reduction is already substantial and further reductions might lead to diminishing returns.

## Conclusion

We have shown that the use of a residual U‐Net for enhancement of LD contrast‐enhanced MRA improved image quality and diagnostic confidence and provided accurate vessel measurements. Enhanced LD images were comparable to HD images in terms of SNR, CNR, edge sharpness, perceptual contrast, agreement of vessel diameters, and diagnostic confidence. Thus, we believe that this technique may enable LD‐MRA to be used in clinical practice without sacrificing clinical utility.

## Supporting information


**Table S1.**Image quality for the prospective cohort.
**Table S2.** Diagnostic accuracy.
**Table S3.** Diagnostic confidence.
**Figure S1.** Bland‐Altman plots of agreement for ascending aorta (AAO), descending aorta (DAO), main pulmonary artery (MPA), left pulmonary artery (LPA), right pulmonary artery (RPA). LoA: limits of agreement; CI: confidence interval.
