# Supplementary material for: Reducing Contrast Agent Dose in Cardiovascular MR Angiography with Deep Learning
Source: J Magn Reson Imaging. 2021 Feb 22;54(3):795–805. doi: 10.1002/jmri.27573 (PMC9681557; doi:10.1002/jmri.27573)

Table S1. Image quality for the prospective cohort.

| **Metric** | **Angiogram** | **n** | **LD** | **ELD** | **HD** | **p (LD vs ELD)** | **p (LD vs HD)** | **p (ELD vs HD)** |
| --- | --- | --- | --- | --- | --- | --- | --- | --- |
| **SNR** | Aortic | 20 | 24.4 ± 9.31 | 59.1 ± 21.81 | 56.8 ± 23.55 | <0.05 | <0.05 | 0.680 |
|  | Pulmonary | 20 | 27.1 ± 12.38 | 53.8 ± 17.55 | 50.4 ± 21.34 | <0.05 | <0.05 | 0.580 |
|  | All | 40 | 25.7 ± 10.9 | 56.5 ± 19.7 | 53.6 ± 22.4 | <0.05 | <0.05 | 0.483 |
| **CNR** | Aortic | 20 | 20.9 ± 8.94 | 55.0 ± 21.07 | 53.2 ± 22.96 | <0.05 | <0.05 | 0.728 |
|  | Pulmonary | 20 | 23.2 ± 12.12 | 49.9 ± 17.36 | 46.8 ± 20.69 | <0.05 | <0.05 | 0.611 |
|  | All | 40 | 22.0 ± 10.6 | 52.4 ± 19.2 | 50.0 ± 21.8 | <0.05 | <0.05 | 0.533 |
| **Edge sharpness** | Aortic | 50 | 0.311 ± 0.0804 | 0.544 ± 0.1393 | 0.551 ± 0.1635 | <0.05 | <0.05 | 0.733 |
|  | Pulmonary | 50 | 0.308 ± 0.1225 | 0.457 ± 0.1897 | 0.454 ± 0.1743 | <0.05 | <0.05 | 0.916 |
|  | All | 100 | 0.309 ± 0.108 | 0.492 ± 0.176 | 0.493 ± 0.176 | <0.05 | <0.05 | 0.930 |
| **Perceptual sharpness** | Aortic | 50 | 2.58 ± 0.914 | 3.67 ± 0.735 | 3.96 ± 0.600 | <0.05 | <0.05 | <0.05 |
|  | Pulmonary | 50 | 2.39 ± 0.894 | 3.14 ± 0.763 | 3.42 ± 0.684 | <0.05 | <0.05 | <0.05 |
|  | All | 100 | 2.47 ± 0.905 | 3.36 ± 0.795 | 3.63 ± 0.703 | <0.05 | <0.05 | <0.05 |
| **Perceptual contrast** | Aortic | 50 | 2.24 ± 0.799 | 4.05 ± 0.684 | 4.07 ± 0.618 | <0.05 | <0.05 | 0.876 |
|  | Pulmonary | 50 | 2.17 ± 0.838 | 3.43 ± 0.777 | 3.56 ± 0.853 | <0.05 | <0.05 | 0.086 |
|  | All | 100 | 2.20 ± 0.822 | 3.68 ± 0.801 | 3.76 ± 0.806 | <0.05 | <0.05 | 0.132 |

Note: SNR and CNR were evaluated in one slice per patient. Edge sharpness, perceptual sharpness and perceptual contrast were evaluated for each vessel. SNR: signal-to-noise-ratio; CNR: signal-to-noise ratio; LD: low-dose; ELD: enhanced low-dose; HD: high-dose.

Table S2. Diagnostic accuracy.

| **Condition** | **n** | **TP** | **Sensitivity** | | **Specificity** | | ***p*-value** |
| --- | --- | --- | --- | --- | --- | --- | --- |
|  |  |  | **LD** | **ELD** | **LD** | **ELD** |  |
| **Aortic coarctation** | 20 | 1 | 1.000 (0.025 ‒ 1.000) | 1.000 (0.025 ‒ 1.000) | 1.000 (0.824 ‒ 1.000) | 1.000 (0.824 ‒ 1.000) | 1.000 |
| **Aortic dilatation** |  | 11 | 0.818 (0.482 ‒ 0.977) | 0.909 (0.587 ‒ 0.998) | 0.778 (0.400 ‒ 0.972) | 1.000 (0.664 ‒ 1.000) | 1.000 |
| **Abnormal arch anatomy** |  | 1 | 1.000 (0.025 ‒ 1.000) | 1.000 (0.025 ‒ 1.000) | 0.789 (0.544 ‒ 0.939) | 0.947 (0.740 ‒ 0.999) | 0.248 |
| **MPA stenosis** |  | 1 | 0.000 (0.000 ‒ 0.975) | 0.000 (0.000 ‒ 0.975) | 1.000 (0.805 ‒ 1.000) | 1.000 (0.805 ‒ 1.000) | 1.000 |
| **LPA stenosis** |  | 0 | NA (0.000 ‒ 1.000) | NA (0.000 ‒ 1.000) | 0.900 (0.683 ‒ 0.988) | 0.950 (0.751 ‒ 0.999) | 1.000 |
| **RPA stenosis** |  | 3 | 1.000 (0.292 ‒ 1.000) | 1.000 (0.292 ‒ 1.000) | 1.000 (0.805 ‒ 1.000) | 0.882 (0.636 ‒ 0.985) | 0.480 |
| **Aortic (all)** |  | 13 | 0.846 (0.546 ‒ 0.981) | 0.923 (0.640 ‒ 0.998) | 0.872 (0.743 ‒ 0.952) | 0.979 (0.887 ‒ 0.999) | 0.289 |
| **Pulmonary (all)** |  | 4 | 0.750 (0.194 ‒ 0.994) | 0.750 (0.194 ‒ 0.994) | 0.963 (0.873 ‒ 0.995) | 0.944 (0.846 ‒ 0.988) | 1.000 |
| **All** | 40 | 17 | 0.824 (0.566 ‒ 0.962) | 0.882 (0.636 ‒ 0.985) | 0.921 (0.850 ‒ 0.965) | 0.960 (0.902 ‒ 0.989) | 0.546 |

Note: Sensitivity and specificity are shown by angiogram and lesion type. Sensitivity for LPA stenosis could not be estimated because none of the patients were positive for this condition. TP: true positives; NA: not available; LD: low-dose; ELD; enhanced low-dose; MPA, LPA, RPA: main, left, right pulmonary arteries.

Table S3. Diagnostic confidence.

| **Condition** | **n** | **TP** | **Confidence** | | | ***p*-values** | | |
| --- | --- | --- | --- | --- | --- | --- | --- | --- |
|  |  |  | **LD** | **ELD** | **HD** | **LD vs ELD** | **LD vs HD** | **ELD vs HD** |
| **Aortic coarctation** | 20 | 1 | 2.85 ± 0.366 | 2.95 ± 0.224 | 2.95 ± 0.224 | 1.000 | 1.000 | 1.000 |
| **Aortic dilatation** |  | 11 | 2.50 ± 0.761 | 2.85 ± 0.366 | 2.90 ± 0.308 | <0.05 | 0.061 | 0.773 |
| **Abnormal arch anatomy** |  | 1 | 2.60 ± 0.754 | 2.90 ± 0.308 | 3.00 ± 0.000 | 0.189 | 0.160 | 0.346 |
| **MPA stenosis** |  | 1 | 2.70 ± 0.657 | 2.70 ± 0.657 | 2.75 ± 0.639 | 1.000 | 1.000 | 1.000 |
| **LPA stenosis** |  | 0 | 2.85 ± 0.366 | 2.90 ± 0.308 | 3.00 ± 0.000 | 0.773 | 0.447 | 0.692 |
| **RPA stenosis** |  | 3 | 2.80 ± 0.410 | 2.80 ± 0.523 | 2.90 ± 0.308 | 1.000 | 1.000 | 1.000 |
| **Aortic (all)** |  | 13 | 2.65 ± 0.659 | 2.90 ± 0.303 | 2.95 ± 0.220 | <0.05 | <0.05 | 0.299 |
| **Pulmonary (all)** |  | 4 | 2.78 ± 0.490 | 2.80 ± 0.514 | 2.88 ± 0.415 | 0.777 | 0.197 | 0.304 |
| **All** | 40 | 17 | 2.72 ± 0.582 | 2.85 ± 0.423 | 2.92 ± 0.333 | <0.05 | <0.05 | 0.064 |

Note: Diagnostic confidence is shown by angiogram and lesion type. TP: true positives; LD: low-dose; ELD; enhanced low-dose; HD: high-dose; MPA, LPA, RPA: main, left, right pulmonary arteries.

Figure S1. Bland-Altman plots of agreement for ascending aorta (AAO), descending aorta (DAO), main pulmonary artery (MPA), left pulmonary artery (LPA), right pulmonary artery (RPA). LoA: limits of agreement; CI: confidence interval.


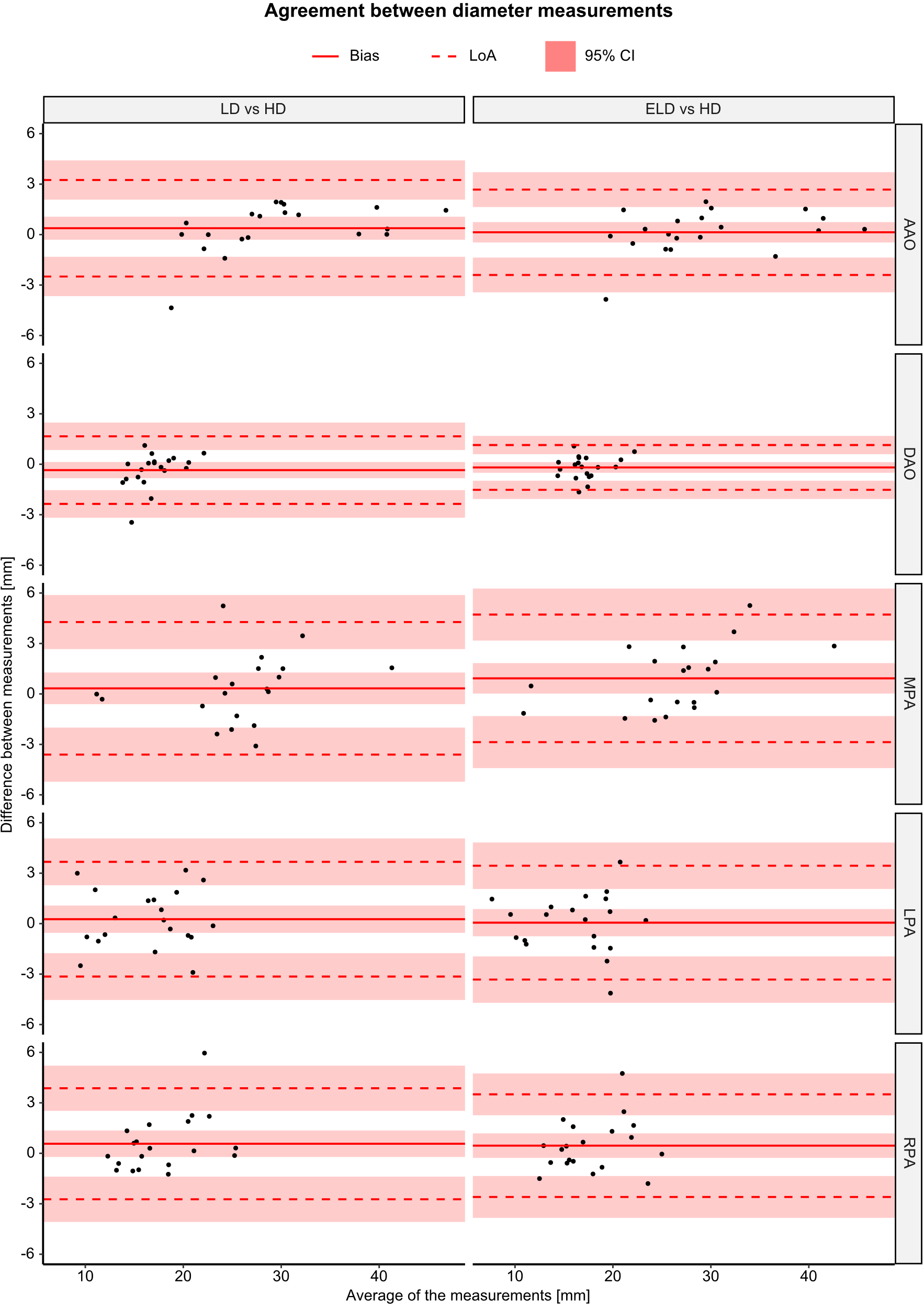

Supplement: Supplementary file 1 — Table S1.Image quality for the prospective cohort. Table S2. Diagnostic accuracy. Table S3. Diagnostic confidence. Figure S1. Bland‐Altman plots of agreement for ascending aorta (AAO), descending aorta (DAO), main pulmonary artery (MPA), left pulmonary artery (LPA), right pulmonary artery (RPA). LoA: limits of agreement; CI: confidence interval. [file JMRI-54-795-s001.docx]
